# Systematic THz study of the substrate effect in limiting the mobility of graphene

**DOI:** 10.1038/s41598-021-87894-5

**Published:** 2021-04-22

**Authors:** Samantha Scarfe, Wei Cui, Adina Luican-Mayer, Jean-Michel Ménard

**Affiliations:** Department of Physics, University of Ottawa, Ottawa, Canada

**Keywords:** Graphene, Electronic properties and devices, Infrared spectroscopy, Electronic properties and materials

## Abstract

We explore the substrate-dependent charge carrier dynamics of large area graphene films using contact-free non-invasive terahertz spectroscopy. The graphene samples are deposited on seven distinct substrates relevant to semiconductor technologies and flexible/photodetection devices. Using a Drude model for Dirac fermions in graphene and a fitting method based on statistical signal analysis, we extract transport properties such as the charge carrier density and carrier mobility. We find that graphene films supported by substrates with minimal charged impurities exhibit an enhanced carrier mobility, while substrates with a high surface roughness generally lead to a lower transport performance. The smallest amount of doping is observed for graphene placed on the polymer Zeonor, which also has the highest carrier mobility. This work provides valuable guidance in choosing an optimal substrate for graphene to enable applications where high mobility is required.

## Introduction

Graphene, an atomic layer of carbon atoms arranged in a honeycomb lattice, is not only a rich playground for uncovering new physical phenomena, but also a potential component in future electronic and optoelectronic technologies^1–3^. For example, graphene has already been implemented into a broadband image sensor array for enhanced photodetection^4^ as well as memristors based on layered two-dimensional materials^5^. Graphene was also demonstrated to be a sensitive chemical and biological sensor^6–8^. With the development of large-scale production techniques such as epitaxial growth or chemical vapor deposition (CVD), large area graphene shows increasing promise for implementation into macroscale modern devices^9^.

Among its many properties, the high mobility of the massless Dirac fermions in graphene plays a key role in the implementation of high-speed electronic devices. Therefore, it is imperative to develop better understanding and control over the factors that affect scattering mechanisms in graphene films. Factors that limit large-area graphene’s mobility can be either intrinsic or extrinsic^10^. Intrinsic limiting factors include scattering from lattice defects, grain boundaries^11–14^, or phonons^15,16^. Due to the formation of grain boundaries and defects during the CVD growth, such scattering mechanisms could be more pronounced in large-area graphene compared to micron-scale mechanically exfoliated samples^17,18^. Among extrinsic limiting factors, scattering due to charged impurities is known to play a significant role^19^. Charged impurities can be present in the underlying substrate^20^, trapped between the film and substrate^21^, or on the surface of graphene in fabrication residues^22^.

Graphene supported by common substrates such as SiO_2_, Si_3_N_4_, quartz or silicon is reported to have carrier mobilities varying between 500 and 10,000 cm^2^V^−1^ s^−1^^19,23–25^. Efforts to engineer substrates that minimize the presence of trapped charges and mechanical deformations led to three orders of magnitude improvement, with the highest mobilities (larger than 10^6^ cm^2^/(Vs)) reported in freely suspended devices^26,27^ or on top of atomically flat boron nitride (BN) crystals^17,28^. It is therefore critical that the graphene-substrate interaction be well understood for successful integration of large-area graphene films with high mobility. Substrates that ensure high mobility for graphene films could be exploited for improving device performance in future graphene integrated electronics.

To date, graphene-substrate interactions have been studied in devices by measuring the unpumped/non-excited carrier dynamics with both optical techniques and electrical transport techniques. For example, far-infrared spectroscopy (3–16 THz) was used to characterize graphene on different polar dielectric and organic polymer films. Graphene on hexamethyldisilazane (HMDS) notably exhibits a mobility four times higher than on SiO_2_^29,30^. Electrical transport was also used to investigate large-scale graphene deposited on several dielectrics commonly used in the semiconductor industry. The highest mobility was achieved when graphene was placed on Si_3_N_4_, which outperformed SiO_2_, HfO_2_, Al_2_O_3_, and tetraethyl orthosilicate. This work indicated that carrier density fluctuation caused by the substrates is one of the main contributing factors for mobility degradation^31^. Other studies relied on time-resolved THz spectroscopy to explore the substrate effect on graphene placed on sapphire, silicon, germanium, polyethylene naphthalate (PEN), polyethylene terephthalate (PET), and quartz^32,33^. This technique provided information about the graphene DC conductivity, but the experimental spectral bandwidth, limited to frequencies up to 3 THz, could not resolve features attributed to carrier scattering. As a result, no information was obtained on carrier density or mobility. Finally, there has been more work that measured^23,34–36^ and even spatially resolved^24,25,37^ the transport properties of wafer scale graphene using THz spectroscopy. These studies, however, are restricted to only one specific substrate, or do not investigate the substrate dependent dynamics at play.

Here, we perform time-domain terahertz transmission spectroscopy over a bandwidth contained within 0.5 to 5.5 THz to measure graphene’s transport properties when supported by substrates with different charged impurity densities, dielectric constants, and surface roughness. Our THz spectral window, extending beyond the limit of most commercial systems, is sufficiently broad to capture the spectral-dependent change in conductivity associated to the Drude roll-off frequency and therefore allows for measurement of both carrier density and Drude mobility. In comparison to other characterization methods based on electrical measurements, THz spectroscopy is a non-invasive technique that does not alter the graphene structure or rely on the deposition of electrical contacts, which composition and configuration often affect the measured transport parameters^38^. Moreover, the THz technique is sensitive to the Drude mobility instead of the field effect mobility. For electrical measurements, the measured field effect mobility and conductivity is affected by surface defects such as grain boundaries, wrinkles and bubbles. Time-domain THz spectroscopy is sensitive to the microscopic conductivity averaged over the distance travelled by an electron during one oscillating cycle of the optical field^11,25^. Since this length typically corresponds to few tens of nanometers in graphene^11^, while defects occur on the micrometer, or even millimeter scale, the Drude mobility and conductivity obtained with THz spectroscopy is minimally sensitive to large scale surface imperfections. Previous works compared the THz technique with other characterization methods relying on van der Pauw structures^25^, back gate measurements^32^, a micro four-point probe^34,39^, or micro Raman spectroscopy^39^. These studies confirmed the potential of THz spectroscopy to remotely map the electrical properties of large graphene sheets and optimize the integration of graphene into electronics or opto-electronics devices^11,24,25,37^.

The selection of substrates in this work is guided by the promise for graphene to be implemented into “beyond Moore” technologies^1^. We compare the mobility of graphene deposited on different materials: two gate dielectrics (SiO_2_ and Si_3_N_4_) which are traditional materials for computational-based applications, silicon with three doping concentrations, and a hydrophobic, flexible, transparent polymer (Zeonor). We find that substrates with minimal amount of charged impurities and substrates that are flat generally exhibit the highest carrier mobilities. Notably, graphene on high-purity silicon and insulating Zeonor outperform the popular gate dielectrics. Our results guide the field of graphene integrated technologies, by providing valuable insights into the transport potential of graphene supported by substrates commonly used in devices.

## Results and discussion

**Figure 1:**
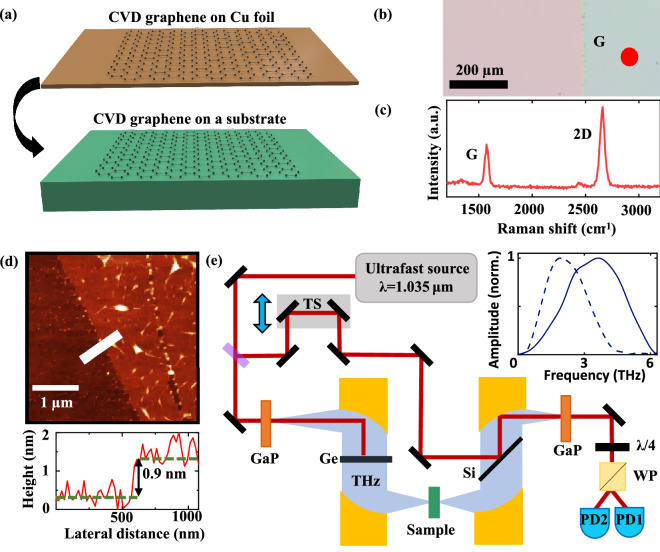
Sample fabrication and experimental set-up. (**a**) Schematic of sample fabrication: CVD graphene on Cu foil is transferred onto a desired substrate. (**b**) 5X optical microscope image of graphene (right section) on SiO_2_ (left section). (**c**) Raman spectrum of a transferred graphene film featuring distinctive G and 2D peaks. (**d**) Atomic force micrograph of graphene transferred on SiO_2_ together with a height profile across the graphene edge, indicated by the white line. (**e**) Schematic of the time-domain terahertz spectrometer with its optical components: beam splitter (BS), translation stage (TS), gallium phosphide crystals (GaP), germanium and silicon wafer (Ge and Si), quarter wave plate (QWP), Wollaston prism (WP), and balanced photodetectors (PD).

**Figure 2:**
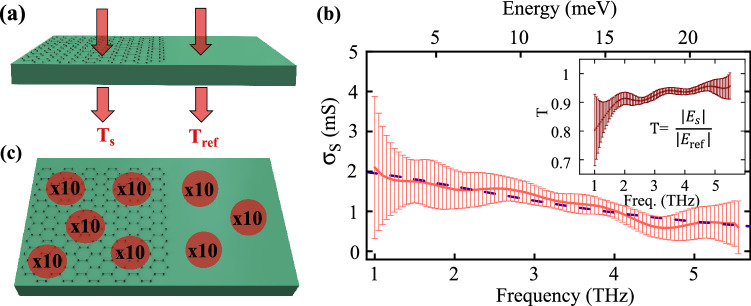
Description of the THz spectroscopy experimental procedure. (**a**) Geometry of the THz reference and sample measurement. (**b**) The real sheet conductivity is extracted by comparing the transmission through the reference and sample (inset). Solid line represents experimental data and the error; dashed line represents the error weighted Drude fit to the data. This data is obtained for graphene on the Si_3_N_4_ substrate. (**c**) Description of the data collected for signal averaging and estimation of uncertainty: 10 scans are taken at a single spot on the graphene sample, indicated by a red dot. This is repeated across the sample at different spots at least 5 times, indicated by the several red dots.

In this work all samples are identically prepared, using graphene synthesized by CVD on Cu foil and transferred onto a substrate as schematically represented in Fig. 1a. A micrograph of transferred graphene on SiO_2_ in Fig. 1b shows continuous coverage across a large area. Atomic force microscopy (AFM) and Raman spectroscopy confirmed monolayer thickness as demonstrated by the height profile (~ 1 nm) and Raman spectrum (I_2D_/I_G_ = 1.94) shown in Fig. 1c,d. For the measurements, we employ a home-built time-domain terahertz spectrometer, schematically represented in Fig. 1e. More details on the sample preparation and experimental setup can be found in the Method section and Supplementary Information. Fourier transform of the time-domain data yields the complex field amplitude spectrum $\tilde{E}_{s}$ transmitted through the graphene sample. As shown in Fig. 2a, a reference field amplitude spectrum $\tilde{E}_{{{\text{ref}}}}$ is collected through the bare substrate, on a region without graphene, to calculate the complex transmission $\tilde{T} = \tilde{E}_{s}$ /$\tilde{E}_{{{\text{ref}}}}$. Each measurement is repeated 10 times (Fig. 2c) under the same experimental conditions to obtain (1) an averaged signal with reduced noise and (2) a frequency-dependent standard deviation indicative of the scan-to-scan experimental error.

**Figure 3:**
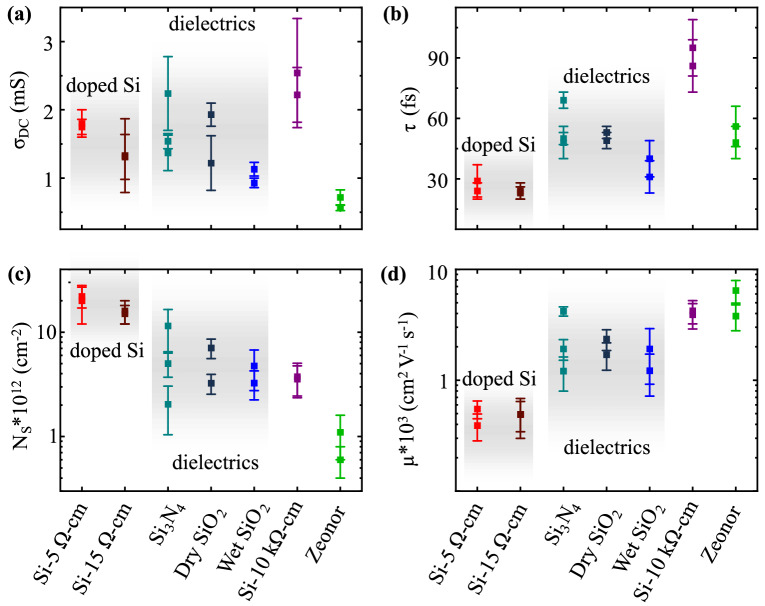
Results obtained for seven distinct substrates by applying the Drude fit to our experimental conductivity spectra. The calculated transport parameters are: (**a**) DC conductivity *σ*_*DC*_, (**b**) scattering time *τ*, (**c**) carrier density *N*_*S*_, and (**d**) mobility *μ*. Solid squares represent the average value measured across several spots on a sample. Error bars represent one standard deviation. Measurements are repeated at least twice for each sample on a different day under similar experimental conditions.

**Figure 4:**
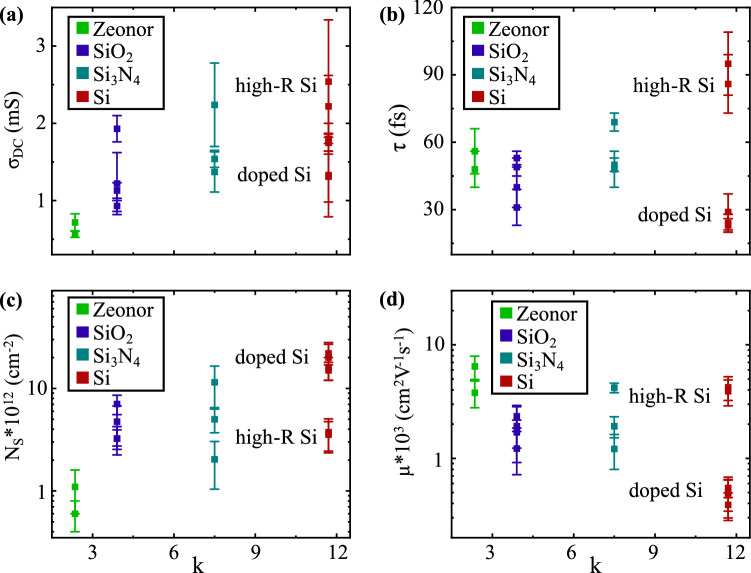
Experimentally measured transport parameters plotted as a function of the substrate dielectric constant κ: (**a**) DC conductivity *σ*_*DC,*_ (**b**) scattering time *τ*, (**c**) carrier density *N*_*S*_, and (**d**) mobility *μ*.

**Figure 5:**
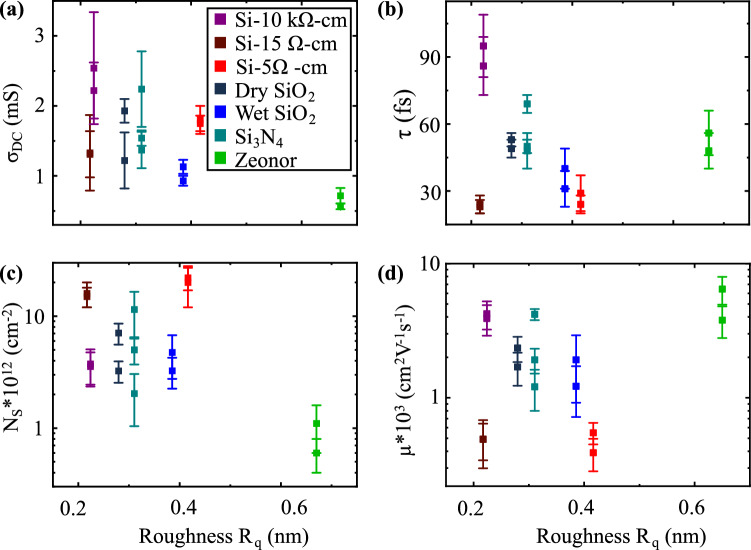
Experimentally measured transport parameters plotted as a function of the substrate roughness R_q_: (**a**) DC conductivity *σ*_*DC,*_ (**b**) scattering time *τ*, (**c**) carrier density *N*_*S*_, and (**d**) mobility *μ*.

The complex sheet conductivity $\tilde{\sigma }_{s}$ can be straightforwardly extracted from $\tilde{T}$ considering graphene as an infinitely thin conducting film^11^, such that:

1 $$\tilde{\sigma }_{s} = \frac{{1 + \tilde{n}_{s} }}{{Z_{0} }}\left( {\frac{1}{{\tilde{T}}} - 1} \right),$$

where $\tilde{n}_{s}$ is the substrate refractive index and $Z_{0}$ the vacuum impedance (376.6 Ω). Figure 2b shows a typical measurement and the corresponding transmission amplitude in the inset with error bars representing scan-to-scan uncertainties. Although Eq. 1 contains complex values, we do not consider the imaginary part of the conductivity in our analysis since this value can induce systematic errors as it is extremely sensitive to local variations of the substrate thickness. Therefore, we consider only the real part of the conductivity $\sigma_{S}$, which has proven to be sufficient to extract reliable graphene properties^25,40,41^. The frequency-dependent Drude model, which has been broadly used to describe carrier intra-band scattering behavior in graphene^24,25,34–36^, yields:

2 $$\sigma_{S} = \frac{{\sigma_{DC} }}{{1 + \omega^{2} \tau^{2} }},$$

where *σ*_DC_ is the DC conductivity, ω is the radial frequency, and *τ* is the scattering time. As a result, we are able to extract values for both *σ*_DC_ and *τ* from the THz data. In general, the analysis of time-domain THz measurements is restricted to data points within a carefully selected spectral window centered around the maximum THz signal, where experimental noise is considered negligible. Here, we use instead a scan-to-scan statistics to include a larger bandwidth while error bars objectively determine the precision of each spectral data point. More specifically, experimental data are fitted to the Drude model (Eq. 2) with a weighting factor inversely proportional to the square of the frequency-dependent uncertainty. This quantitative approach ultimately allows more data points to be considered in the analysis and can therefore improve accuracy and reliability. To determine another type of uncertainty related to sample anisotropy, we collect data on more than 5 different positions on each sample and perform again the analysis described above (Fig. 2c). We rely on these multiple measurements and their standard deviation to evaluate a relative uncertainty of *σ*_DC_ and *τ*, which is, in turn, used to calculate the relative uncertainty on other carrier transport parameters displayed in Figs. 3, 4 and 5. Finally, the full data acquisition process on each graphene sample is repeated at least twice on different days to test the reproducibility of our THz spectroscopy technique. This last step provides at least two data points, with their respective error bar, for all measured transport parameters and each substrate considered in our study.

We use the experimental procedure described above to obtain the parameters *σ*_DC_ and *τ* of graphene samples deposited on seven different substrates: three gate dielectrics on Si wafer (Si_3_N_4_, wet thermal grown SiO_2_, and dry thermal grown SiO_2_), Si with three doping concentrations corresponding to a resistivity of 10 kΩ cm (intrinsic doping), 15 Ω cm (p-doped), and 5 Ω cm (p-doped), and finally the flexible polymer Zeonor, a cyclo-olefin copolymer. We note that all bare substrates display high transparency to our broadband THz probe. Assuming a diffusive Drude-type transport regime^42–45^, we can then calculate graphene’s carrier concentration (*N*_*S*_) and the mobility (*μ*) from the extracted *σ*_DC_ and *τ* using the following relations:

3 $$N_{S} = \frac{{\pi \hbar^{2} }}{{v_{F}^{2} e^{4} }}\left( {\frac{{\sigma_{DC} }}{\tau }} \right)^{2} ,$$

4 $$\mu = \frac{{\sigma_{DC} }}{{eN_{s} }} = \frac{{v_{F}^{2} e^{3} }}{{\pi \hbar^{2} }}\frac{{\tau^{2} }}{{\sigma_{DC} }} = \frac{{v_{F} e}}{\sqrt \pi \hbar }\frac{\tau }{{\sqrt {N_{S} } }},$$

where the Fermi velocity for graphene *v*_*F*_≈ 10^6^ m/s. As a result, THz measurements enable an effective and non-invasive method for probing the influence of the substrate on the transport properties of graphene films.

In Fig. 3 we plot the calculated parameters *σ*_DC,_ τ, *N*_*S*_ and $\mu$ for the seven different substrates described above. Figure 3c shows that the carrier concentration *N*_S_ in graphene is strongly dependent on the substrate. This variation, by more than an order of magnitude, can be attributed to the densities of free and trapped charges in the substrate, which have previously been identified as two dominant parameters affecting *N*_S_^19,43^_._ For the insulating substrates, trapped charges are particularly relevant, as those present at the surface may directly transfer charges to graphene and those beneath the surface may still capacitively induce charges in graphene. For the conductive substrates, *N*_S_ is largely due to direct charge transfer. In our experiment, the largest values of *N*_*S*_ are obtained for graphene placed on doped Si and approach 10^13^ cm^−2^, corresponding to graphene’s Fermi level being 350 meV away from the Dirac point. This value is comparable to epitaxial graphene grown on SiC^46^. Insulating substrates, including typical gate dielectrics (SiO_2_ and Si_3_N_4_), high-resistivity Si (*ρ* = 10^4^ Ω cm) and the polymer Zeonor (*ρ* = 10^16^ Ω cm), lead to a lower *N*_S_. The lowest doping level we measured corresponds to graphene on Zeonor (*N*_S_ = 8 × 10^11^ cm^−2^), indicating that this substrate has a minimal amount of free and trapped charges contributing to the doping of the 2D material on its surface.

Figure 3d shows the calculated mobilities for graphene films placed on the different substrates, presented in increasing order from left to right based on the calculated *N*_S_. We obtain higher mobilities for less doped samples, consistent with the notion that substrate charged impurities create an electrostatic potential that limits the mobility^19,20^. Interestingly, graphene on high-resistivity Si shows a higher mobility than the one on gate dielectric substrates although these samples have a similar carrier concentration *N*_S_. This result can be attributed to a more uniform electrostatic potential profile at the surface of the high-resistivity Si substrate, which results in a longer scattering time *τ* and, according to Eq. (4), an increased mobility. The shorter τ observed with the SiO_2_ and Si_3_N_4_ substrates may be caused by a non-uniform distribution of trapped charges creating a rough electrostatic potential or by a larger density of these trapped charges increasing the number of electron hole puddles in graphene^47^.

The dielectric constant κ of the substrate or environment can play an important role in the transport properties of graphene; larger values of κ imply the ability of an environment to readily screen charged potentials created by trapped scattering centers, and therefore enhance the scattering time and, consequently, the carrier mobility^48–50^. In Fig. 4, we plot the measured transport parameters as a function of the substrate’s dielectric constant *κ*. For the calculated *τ* and *μ* in Fig. 4b,d, the value obtained for graphene placed on the doped Si substrate slightly departs from the general trend. We attribute this discrepancy to a strong inhomogeneous electrostatic potential created by the boron dopants in these substrates. Surprisingly, we observe that graphene on Zeonor exhibits a relatively long scattering time (*τ* = 52 fs) and the highest mobility, even though this substrate has the lowest dielectric constant among the samples measured. This indicates that the screening of charged impurities in the substrate is not necessarily the main contributor to the high mobility, but other factors come into play, such as the density of trapped and free charges in the substrate, as discussed previously. In general, Fig. 4 does not show a clear relationship between κ and the measured parameters characterizing the transport properties. This suggests that, among the variety of factors demonstrated to influence the electronic properties of the graphene films in our experiment, the dielectric constant of the substrate does not appear to play the key role.

Although much less systematically studied, substrate roughness can also affect the carrier mobility in graphene since a smooth interface contributes to reduced strain of the film or eliminates wrinkles and edges that could induce scattering. Using atomic force microscopy, we measure the roughness (*R*_q_) of the seven substrates used in this experiment (Fig. 5). As shown in Fig. 5b, we observe longer scattering times for smooth substrates (small values of *R*_q_) and vice-versa. For example, we measure *τ* = 91 fs for Si-10 kΩ cm (*R*_q_ = 220 pm) while *τ* = 36 fs for wet SiO_2_ (*R*_q_ = 380 pm). However, we note two exceptions in our experiment: (1) graphene on doped Si features a short scattering time although the substrate is relatively smooth. This suggests that Coulomb scattering could be a dominant mechanism in this case. (2) At the other extreme, graphene on Zeonor has a relatively long scattering time while the substrate roughness is higher, *R*_q_ = 650 pm. To assess the adhesion of graphene to Zeonor, we measured the roughness of the graphene sheet on Zeonor, which shows a smoother morphology (*R*_q _= 520 pm), suggesting relatively poor adhesion of the graphene to the substrate. As a result, graphene may experience a reduced influence from the substrate roughness. Note that the mobility in Fig. 5d, which is intrinsically related to the scattering time via Eq. (4), also shows a similar trend than the one discussed above. The outperformance of graphene on a hydrophobic polymer compared to a gate dielectric is consistent with previous reports where graphene field effect transistors on parylene^51^ and HMDS^29,30^ demonstrated reduced ambient doping and hysteresis compared to those on SiO_2_.

## Conclusion

In summary, we have demonstrated the implementation of terahertz transmission spectroscopy for probing the equilibrium transport properties of CVD graphene supported by seven substrates, distinct in their density of charged impurities, dielectric constant and surface roughness. We find that the resulting conductivity spectra for graphene films agree well to a Drude model for intraband behavior, consistent with other reports. We have demonstrated a method for weighted Drude fitting based on statistical averaging and extracted transport properties (*N*_*S*_*, μ*, *τ*, and *σ*_DC_), for graphene supported by distinct substrates. When comparing the results on different substrates, we find the highest mobility for graphene placed on pure silicon and the polymer Zeonor, which outperformed commonly used gate dielectrics. This highlights that the densities of trapped and free charged impurities in the substrate are likely key limiting factors of the graphene film’s mobility. We also find the mobility of devices to be generally improved for substrates that are smoother, except for graphene on Zeonor. In this case, we interpret the high mobility to be due to both the poor adhesion between Zeonor and graphene, minimizing the substrate effect, and to the low density of trapped charged impurities at the interface. A flexible polymer, therefore, can be considered as a promising alternative to some gate dielectrics. These results provide valuable guidance for the effective integration of graphene into technological platforms where high mobility is necessary.

## Methods

### Fabrication of graphene devices

We use commercially available CVD graphene, on copper foils (source: Graphenea), which is transferred onto a substrate using a standard PMMA-based wet etching technique^52,53^. We first remove any graphene residues on the backside of the copper foil with an oxygen plasma etch while the graphene sheet on the frontside is protected by a top-layer of PMMA. The copper foil is then dissolved in chemical etchant, leaving a floating graphene-PMMA film that we rinse under water and deposit on a substrate. Finally, the PMMA is dissolved in a bath of acetone to obtain a graphene sheet on substrate. A more detailed description of the transfer process is outlined in the Supplementary Information.

### THz spectroscopy measurement

The time-resolved THz system is based on a near-infrared source delivering 180 fs pulses at 1035 nm, which can be compressed to ~ 50 fs pulses using spectral broadening in a gas-filled hollow-core photonic crystal fiber and dispersion compensation optics^54^. These pulses are focused onto a nonlinear GaP crystal where THz transients are generated by difference frequency mixing. The time-domain detection relies on electro-optic sampling inside a second GaP crystal^55^. The measurements are performed in a transmission geometry. Apodization of the time-domain signal allows us to exclude Fabry-Pérot echoes. Therefore, only the pulse directly transmitted through the sample is considered in extracting the graphene transport parameters. The samples, at room temperature and under normal ambient conditions, are placed at the focus of an off-axis parabolic mirror, with a numerical aperture NA = 0.5, focusing the THz beam to a 400 µm diameter (1/e^2^) at 1 THz. The optical probe size is significantly larger than any local defects, such as wrinkles, bubbles and other imperfections revealed by AFM images of the samples. Large scale anisotropy reported in previous work^11,24,25,36,37^, is accounted for by performing measurements at different locations on the sample at a ~ 5 mm separation. Note that we purge the system with dry air (< 0.5% RH) to minimize THz absorption in water vapor.

## Supplementary Information

Supplementary Information
