# Supplementary material for: Systematic THz study of the substrate effect in limiting the mobility of graphene
Source: Sci Rep. 2021 Apr 22;11:8729. doi: 10.1038/s41598-021-87894-5 (PMC8062515; doi:10.1038/s41598-021-87894-5)
Supplement: Supplementary file 1 — Supplementary Information [file 41598_2021_87894_MOESM1_ESM.pdf]

Supplementary Information for 'Systematic THz study of the substrate effect in limiting the mobility of graphene'

Samantha Scarfe, Wei Cui, Adina Luican-Mayer, Jean-Michel Ménard

Department of Physics, University of Ottawa, Ottawa, Canada

Fabrication of graphene devices

In this work, we use commercially purchased CVD graphene on (4 inch × 3 inch) copper foils (source: Graphenea). We cut the large foil into 1 cm x 1 cm pieces for sample transfer. The graphene grown on copper has a layer of PMMA spun on it by the manufacturer but requires a backside oxygen plasma etch before transfer. Graphenea partially removes the backside graphene, however not completely. If it is not removed it can remain beneath the surface, trap molecules of copper foil that will diminish the quality of the final transferred CVD film. To remove the graphene on the backside we place the CVD graphene on copper foils upside down in a reactive ion etcher (RIE) and expose their backside to 10 sccm of O_2 plasma at 100 W for 1 minute at 1.1 Pa (Fig. S1a). The RIE applies plasma from the top down while the desired graphene film is protected by a layer of PMMA. Next, we place the sample in a dish of commercially mixed copper etchant (Sigma Aldrich) on a hot plate set to 70 ^{o}C (Fig. S1b). The elevated temperature increases the etching rate, allowing this step to take between 10 to 60 minutes only. The etch is considered complete when there is visually no copper remaining. The transparent graphene-PMMA film is then lifted out using an etchant resistant spoon and placed into a dish of distilled water for 5 minutes. This is repeated for two more distilled water dishes, each providing an increasingly clean rinse of the copper etchant. After the last water rinse is complete, the desired substrate is introduced into the dish from below and delicately raised towards the graphene-PMMA film at a 45-degree angle (Fig. S1c). The graphene-PMMA film is left in a sample holder inside a fume hood environment for eight hours to allow any water that may have been captured between the sample and substrate to evaporate, and to promote adhesion of the graphene film to the substrate. The PMMA film is dissolved the next day by placing the sample in a bath of warm acetone at 50 ^{o}C for 1 hour (Fig. S1d). The final result is a 1 cm × 1 cm CVD graphene layer supported by its substrate. Some samples used in this work are shown in Fig. S2, where the region covered in graphene is indicated with a dashed line.

For the experiments presented in this manuscript, we used the same transfer technique for all substrates. We previously reported its details[6] and showed that it can ensure minimal device-to-device variation in terms of doping level and surface morphology. As the transfer process inadvertently results in macroscopic bubbles, folds or tears, the quality of the transfer technique plays an important role in determining the transport properties. Note that improvements to the standard wet transfer techniques could be based, for example, on using scanning probes (Rosenberger, M. *et al.*, ACS Appl. Mater. Interfaces, 2018, 10, 10379-10387) or on using polymers as support in the graphene transfer (Hong, J.-Y. *et al.* Adv. Mater., 2016, 28, 2382-2393).

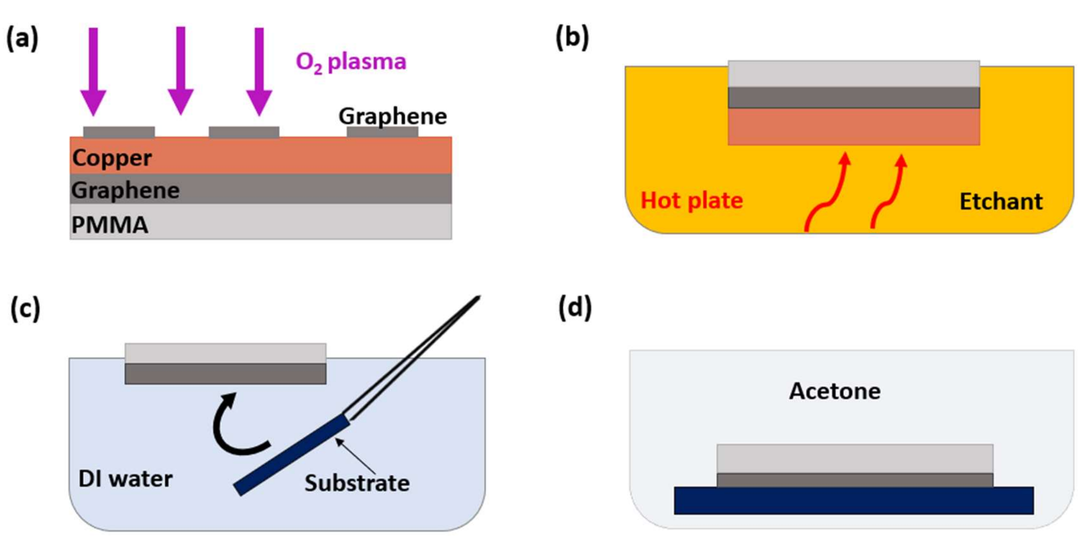

Figure S1: Schematics of the different steps required for the fabrication of a graphene sheet on substrate. (a) Graphene residues on the backside of the copper foil are removed with an oxygen (O2) plasma etch while the graphene sheet on the frontside is protected by a top-layer of PMMA. (b) The copper foil is dissolved in chemical etchant. (c) The graphene-PMMA film floating in rinsing water is placed on the substrate coming from below. (d) The graphene-PMMA film is placed in a dish of acetone to remove PMMA. The sample is left inside a fume hood environment for eight hours to relax and dry.

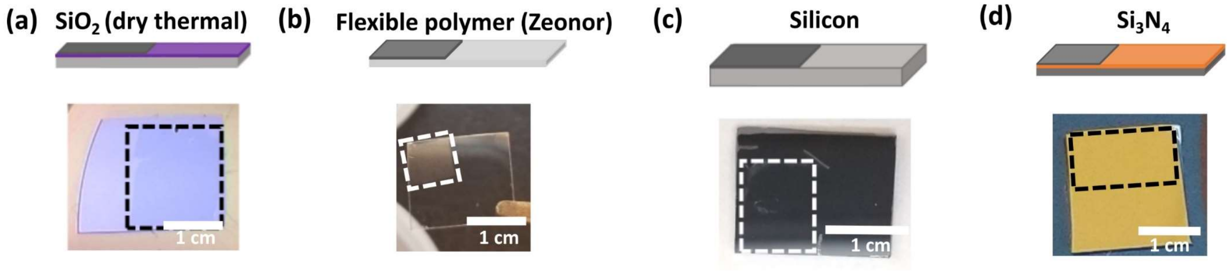

Figure S2: Schematics and photographs of the graphene samples deposited on (a) dry thermally grown SiO_2, (b) Zeonor, (c) silicon, and (d) Si_3N_4.

Terahertz (THz) conductivity spectra and Drude fits

Figure S3 shows conductivity spectra fitted to extract the carrier transport parameters. Each spectrum represents the average conductivity measured across a sample on a given day. We perform weighted fits to the real component of the Drude model to extract the DC conductivity σ_{DC}, carrier scattering time τ, carrier density N_S, and Drude mobility μ.

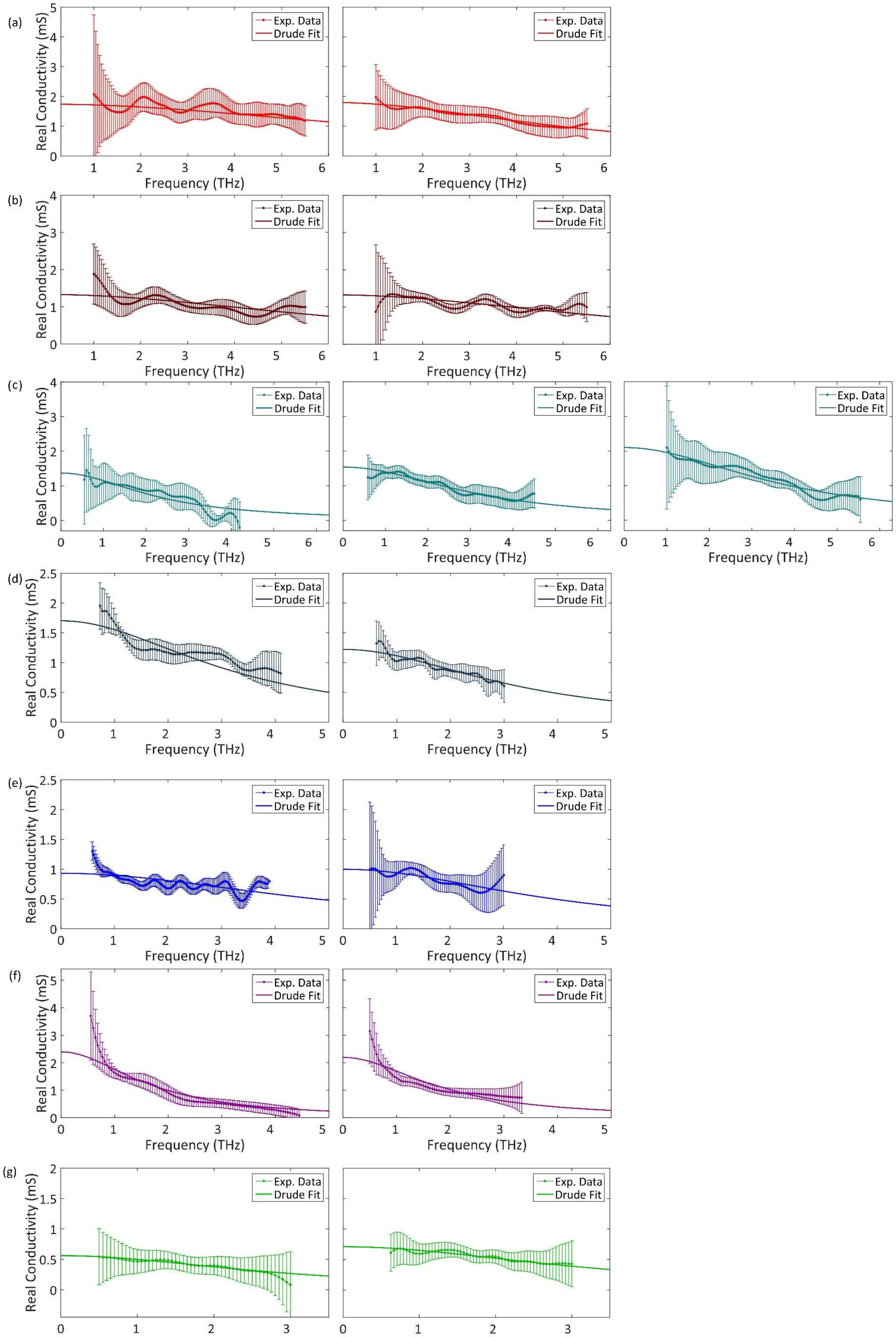

Figure S3: Real part of the conductivity spectrum measured with time-resolved THz spectroscopy for graphene on (a) Si (5 Ω-cm), (b) Si (15 Ω-cm), (c) Si_3N_4, (d) Dry thermally grown SiO_2, (e) wet thermally grown SiO_2, (f) high-resistivity Si (10 kΩ-cm), and (g) Zeonor.

Data comparison

Figure S4 shows good agreement between the measured transport parameters in Fig. 3 (in the main text) and previously reported results obtained on CVD graphene deposited on SiO_2 and Si_3N_4 substrates. We considered four studies relying on a non-contact far-infrared characterization technique, including THz spectroscopy.

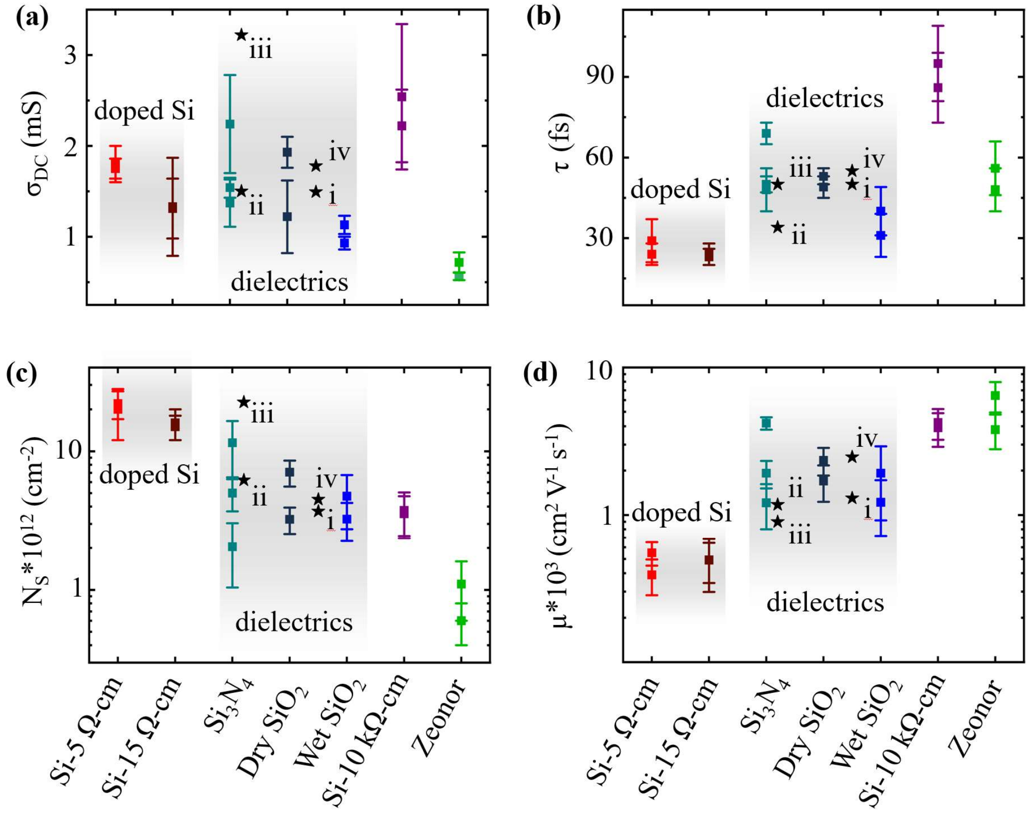

Figure S4. Comparison between transport parameters reported in Fig. 3 (in the main text) showing (a) DC conductivity σ_{DC}, (b) scattering time τ, (c) carrier density N_S, and (d) mobility μ, and previous reports of non-contact optical characterization of CVD graphene deposited on a Si_3N_4 substrate: i) [24] Buron, J. D. *et al. Scientific Reports* **5**, 12305 (2015) and ii) [25] Buron, J. D. *et al. Opics. Express* **23**, 30721 (2015), and on a SiO_2 substrate: iii) [23] Yan, H. *et al. ACS Nano* **5**, 9854 (2011) and iv) [30] Kim, J. Y. *et al. Applied Physics Letters* **98**, 201907 (2011). Note that reported values for *N* and μ were used to calculate τ in i) and ii), and to calculate σ_{DC} in iv).
